# Formalin pre-fixation improves autopsy histology

**DOI:** 10.4322/acr.2021.291

**Published:** 2021-05-27

**Authors:** Jennifer Vazzano, William Sinclair, Bradley Zehr, Patricia Allenby

**Affiliations:** 1 The Ohio State University Wexner Medical Center, Department of Pathology, Columbus, OH, USA

**Keywords:** Pathology, Histology, Autopsy, Quality Improvement, Tissue Fixation

## Abstract

Microscopic findings in key tissues are often critical to determine the cause of death in medical autopsies. The overall quality of histologic sections depends on numerous pre-analytic factors, among which are tissue section size and thickness. We designed a prospective quality improvement study to determine whether a simple intervention of formalin pre-fixation of myocardium, liver, and kidney tissues could improve the ease of cutting and quality of autopsy histologic sections as assessed by histotechnicians and pathologists. Of 46 autopsies included in the study, 21 were randomly assigned to formalin pre-fixation, and 25 underwent routine sectioning without formalin pre-fixation. A significant improvement in overall quality score by histotechnicians was detected in the sections from pre-fixed autopsy tissues compared to the control group (p=0.0327). There was no significant difference in quality score between the two groups as assessed by pathologists. Our autopsy quality improvement study demonstrates that a simple, low-cost intervention of formalin pre-fixation of fresh autopsy tissues for 90 minutes could significantly improve the overall quality of sections submitted for histologic processing.

## INTRODUCTION

The primary goal of a medical autopsy is to collect and analyze clinical and pathologic data to determine the likeliest cause of death and contributing factors. Pathologic data generated from an autopsy include gross anatomic findings, histologic findings, and results from ancillary studies such as postmortem blood testing. Histologic findings are based on pathologist interpretation of slides containing representative sections of key tissues including myocardium, kidney, and liver, among others. Like all clinical laboratory testing, interpretation of histologic sections depends on pre-analytic, analytic, and post-analytic factors.^1^ Among the pre-analytic factors contributing to the quality of histologic analysis of autopsy tissues are the thickness and size of the tissue sections submitted for histologic processing.^2^

Tissue sectioning can be particularly challenging on fresh, non-preserved tissues as occurs in the autopsy setting. At our institution, residents in training perform autopsy dissection and tissue sectioning. Our institution conducts approximately 220 medical autopsies annually, and feedback was received from histotechnicians that the autopsy tissue sections were too thick, causing suboptimal tissue fixation, increased microtomy difficulty, and decreased overall histology quality. The optimal tissue thickness for histologic processing is in the 2 to 3 mm range and no thicker than 3 to 4 mm, which can be technically challenging to produce from fresh tissues.^3^

We hypothesized that pre-fixation of autopsy tissue (firming up the tissue) would help it to be trimmed to thinner and more consistent sections. We conducted a prospective study to compare histology quality between autopsy cases in which tissues had been pre-fixed in formalin prior to sectioning by residents versus cases in which tissues had not undergone formalin pre-fixation. The aim of our study was to determine whether a simple intervention of formalin pre-fixation of tissues during an autopsy could significantly improve the quality of histologic sections as determined by histotechnicians and pathologists.

## METHODS

**Figure 1:**
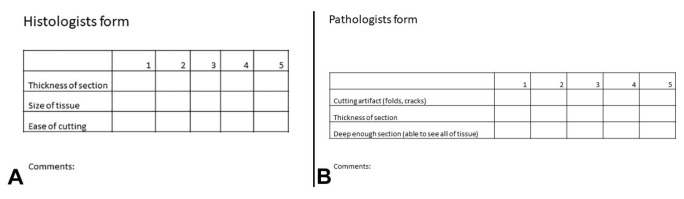
Histologists and Pathologists questionnaires. **A –** The questionnaire sent to histotechnicians to score the thickness, size, and the ease of cutting of the tissue on a 1-5 scale (1 being poor and 5 being best; **B** – The questionnaire sent to pathologists to assess the histologic sections for cutting artifacts, thickness of the section, and depth of the section on a 1-5 scale (1 being poor and 5 being best).

A total of 46 autopsy cases were included in the study from a 9-month period (October 2018 to June 2019). Only autopsy cases involving both thoracic and abdominal cavity examination were included in the study. At our institution, residents in training complete autopsies, including sectioning tissues and placing the sections into histology cassettes, and this workflow was maintained for the study. The resident randomly assigned these cases to one of two groups: formalin pre-fixation or control. In cases assigned to formalin pre-fixation, the resident placed small pieces of the bilateral kidneys, left and right cardiac ventricles, interventricular septum, and liver in formalin containers for approximately 90 minutes while the autopsy was ongoing. After 90 minutes and before autopsy completion, residents sectioned the pre-fixed tissues for placement into histology cassettes. The control cases were processed according to the usual protocol without formalin pre-fixation prior to sectioning, so sections for control cases were taken by residents from fresh tissue and placed directly into histology cassettes. The cassettes were sent for histology processing with an attached questionnaire (“histologists form”) to be completed by the histotechnicians to score the thickness, size, and the ease of cutting of the tissue on a 1-5 scale, with 1 being poor and 5 being best (Figure 1A). The same questionnaire (“histologists form”) was sent with both formalin pre-fixation cases and control cases. A similar questionnaire (“pathologists form”) was provided to the case pathologist at the time of glass slide review to assess the histologic sections for cutting artifacts, thickness of the section, and depth of the section on a 1-5 scale, with 1 being poor and 5 being best (Figure 1B). The same questionnaire (“pathologists form”) was sent with both formalin pre-fixation cases and control cases. A comment section was included on both questionnaires to capture additional qualitative feedback. Both histotechnicians and pathologists were blinded as to the case assignment (formalin pre-fixation or control). All cassettes (for both formalin pre-fixation group and control group) were sent to the same histology lab for processing.

## RESULTS

Of the 46 autopsy cases included in the study, 21 were randomly assigned to the formalin pre-fixation group and 25 to the control group. After excluding cases with missing values (score values missing from either the histotechnician or pathologist questionnaire), 34 cases remained for analysis. For each case, three histotechnician scores and three pathologist scores were available. The explanatory variable was “pre-fixation” and took on two values (0=no, 1=yes). A linear regression model was used to estimate the effect of pre-fixation on evaluations: y= β_0_ + β_1_x + Ɛ, where “y” is the average score of the histotechnician, the average score of the pathologist, or the average score across both histologists and pathologists. A significant relationship was found for the histotechnician ratings, with pre-fixation resulting in an average increase in rating of 0.244 (p=0.0327, multiple R^2^ = 0.1348). Thus, pre-fixation of autopsy tissue resulted in increased overall quality score by histotechnicians. There was no significance found when analyzing any of the 6 score categories separately, the average pathologists’ score (average increase in rating of 0.1684, p=0.541, multiple R^2^=0.01179), or the average score for both the histologists and pathologists (average increase in rating of 0.2064, p=0.192, multiple R^2^= 0.05136). Qualitative feedback from the questionnaire “comment” section indicated that heart sections were still too thick for 3 cases, coronary artery sections were still too thick or incomplete in 3 cases, bone marrow sections were still too thick in 5 cases, deeper sections were still needed in 3 cases, and tissue sections were still too large for the histologists to cut properly in 3 cases.

## DISCUSSION

Autopsy tissue sampling presents special challenges for the diener, histotechnician, and pathologist.^4^ Unlike other surgical pathology specimens, autopsy tissues are typically not formalin-fixed at the time of sampling. The process of refrigeration improves preservation of tissues; however, the decedent’s body habitus and local hospital resources may limit preservation of the organs with this technique. The goal of this study was to identify a reproducible, low-cost, efficient process to improve the quality of histologic sections of autopsy tissues.

**Figure 2:**
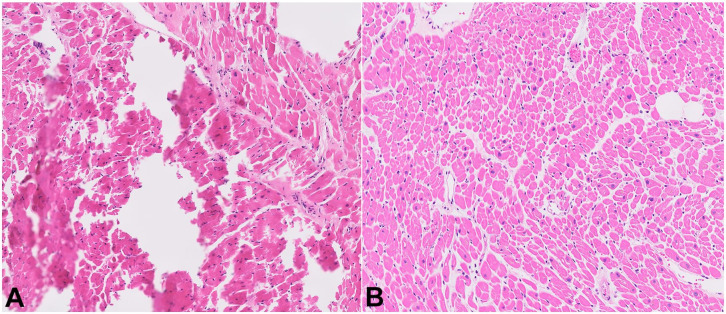
Comparison of control tissue and formalin pre-fixed tissue. **A** – Histology of a tissue section taken from the right heart ventricle demonstrating artifacts; tissue was sectioned fresh without formalin pre-fixation (control); **B –** Improved histology of a tissue section taken from the right heart ventricle after formalin pre-fixation and subsequent tissue sectioning.

At our institution, the relevant tissues are sectioned by residents directly after removal from the body, placed in cassettes, and then fixed in formalin. This process led to artifacts and impaired interpretation of slides (Figure 2A, right ventricle). Naturally, this process is affected by histotechnician and pathologist experience and skill level. However, quality improvement methods have facilitated a way to improve the resulting histology of autopsy specimens, which in turn leads to improved diagnosis and patient care.^2^

Our study demonstrates that pre-fixation of tissues for approximately 90 minutes before sectioning improves the quality of histology sections of the right kidney, left kidney, left ventricle, right ventricle, interventricular septum, and liver (Figure 2B, right ventricle). Histotechnicians at our institution reported significant improvement in the thickness, size, and ease of cutting of the tissue with this pre-fixation process.These results likely represented an improvement in the sectioning of pre-fixed tissues by the pathology resident during the autopsy.

There are many challenges a pathologist, pathology assistant, resident or diener face when sectioning tissues at the time of an autopsy. Those performing the autopsy routinely wear thick, cut-resistant gloves that limit the ability to manipulate small tissue fragments, which can result in larger and thicker sections. Furthermore, the tissue that needs to be carefully sectioned is usually quite soft and often coated by blood or other body fluid. Fixing the tissue for 90 minutes firms up the tissue, makes it easier to manipulate and control, and thus helps it to be trimmed to thinner and more consistent sections.

Although pathologists reported no improvement while interpreting the slides, this may be because the pathologist has additional tools to facilitate interpretation when initial histology is sub-par. For example, if an adequate tissue section was not achieved initially by the histotechnologist, deeper sections are performed, which sometimes leads to an adequate tissue section. However, the need for additional tissue sections leads to extra time and resources spent in the histology lab. This can be avoided with pre-fixation of tissues so the histotechnologist can capture the appropriate tissue section on the first attempt.

One strength of our study is the reproducibility of our findings and the straightforward implementation of our proposed pre-fixation method, which can be used by anyone sectioning autopsy tissue (pathologists, pathology assistants, residents, or dieners). The only materials needed are a container, a method of separating and labeling tissues, and formalin. It is important that the tissues are properly separated and labeled during fixation to avoid confusion. We recommend a clear labeling system on the container and dividers to separate individual tissue fragments. Our institution used a numbering system that corresponded to the designated cassettes for specific tissues. For example, the ‘#2’ on our formalin container indicated that the tissue should later be placed in cassette ‘A2’, which was designated for the left ventricle. In the autopsy suite adjacent to the dissection table, we printed a list of our routine sections that included cassette designations. An additional strength of our study is the blinding of both the histotechnicians and pathologists to which group they were grading.

The study was limited by the relatively small number of cases evaluated. Specifically, fourteen cases were removed from the study because various data points were lacking. Clearer communication with the histotechnologists and pathologists at the beginning of the study could have improved the response rate. Another limitation of the study is that junior pathology residents completed the sectioning of the autopsy tissues; however, because residents took the sections during the entire study, there was no change in experience level. Furthermore, residents are the ones commonly taking autopsy sections in training programs, and autopsies are commonly completed at larger academic hospitals that have regional autopsy centers like ours. This method of pre-fixation resulted in improved ease of cutting by histotechnicians, which was the goal of this study.

## CONCLUSION

In summary, the goal of this quality improvement study was to assess whether a simple intervention of formalin pre-fixation of key autopsy tissues for approximately 90 minutes during autopsy could improve the quality of autopsy histologic sections as assessed by histotechnicians and pathologists. We found that formalin pre-fixation did increase the overall quality score by histotechnicians, but did not affect quality scoring by pathologists at the time of glass slide review. Nevertheless, this low-cost, low-complexity intervention is widely applicable to academic practice settings and easy to implement by anyone (pathologists, pathology assistants, and residents) completing autopsy tissue sectioning to significantly improve the quality of sections sent for histology processing.
